# Risk Identification in Perinatal Health Care Settings via Technology-Based Recruitment Methods: Comparative Study

**DOI:** 10.2196/48823

**Published:** 2024-03-04

**Authors:** Jessica R Beatty, Logan Zelenak, Spencer Gillon, Lucy McGoron, Gregory Goyert, Steven J Ondersma

**Affiliations:** 1 Merrill Palmer Skillman Institute for Child & Family Development Wayne State University Detroit, MI United States; 2 Maternal Fetal Medicine Women's Health Services Henry Ford Health Detroit, MI United States; 3 Charles Stewart Mott Department of Public Health College of Human Medicine Michigan State University Flint, MI United States; 4 Department of Obstetrics, Gynecology, and Reproductive Biology College of Human Medicine Michigan State University East Lansing, MI United States

**Keywords:** participant recruitment, engagement, health care screening, mobile phone

## Abstract

**Background:**

Digital screening and intervention tools have shown promise in the identification and reduction of substance use in health care settings. However, research in this area is impeded by challenges in integrating recruitment efforts into ongoing clinical workflows or staffing multiple study clinics with full-time research assistants, as well as by the underreporting of substance use.

**Objective:**

The aim of the study is to evaluate pragmatic methods for facilitating study recruitment in health care settings by examining recruitment rates and participant characteristics using in-person–based versus flyer approaches.

**Methods:**

This study compared recruitment rates at a Women’s Health clinic in the Midwest under 2 different recruitment strategies: in person versus via a flyer with a QR code. We also examined the disclosure of substance use and risk screener positivity for the 2 strategies. We also obtained information about the current use of technology and willingness to use it for study participation.

**Results:**

A greater percentage of patients recruited in person participated than those recruited via flyers (57/63, 91% vs 64/377, 17%). However, the final number recruited in each group was roughly equal (n=57 vs n=64). Additionally, participants recruited via flyers were more likely to screen positive for alcohol use risk on the Tolerance, Annoyed, Cut Down, Eye-Opener alcohol screen than those recruited at the clinic (24/64, 38% vs 11/57, 19%; *χ*^2^_1_=4.9; *P*=.03). Participants recruited via flyers were also more likely to screen positive for drug use risk on the Wayne Indirect Drug Use Screener than those recruited at the clinic (20/64, 31% vs 9/57, 16%; *χ*^2^_1_=4.0; *P*=.05)*.* Furthermore, of the 121 pregnant women, 117 (96.7%) reported owning a smartphone, 111 (91.7%) had an SMS text message plan on their phone, and 94 (77.7%) reported being willing to receive SMS text messages or participate in a study if sent a link to their phone.

**Conclusions:**

The distribution of flyers with a QR code by medical staff appears to be an efficient and cost-effective method of recruitment that also facilitates disclosure while reducing the impact on clinic workflows. This method of recruitment can be useful for data collection at multiple locations and lead to larger samples across and between health systems. Participant recruitment via technology in perinatal health care appears to facilitate disclosure, particularly when participants can learn about the research and complete screening using their own device at a place and time convenient for them. Pregnant women in an urban Midwestern hospital had access to and were comfortable using technology.

## Introduction

Screening, brief intervention, and referral for treatment (SBIRT) approaches proactively address substance use in primary care settings and potentially reach those at risk, regardless of willingness to seek treatment. Large proportions of at-risk groups can be reached with SBIRT, particularly in the perinatal period where most pregnant women seek prenatal care. The consequent need for proactive screening, together with the promising efficacy of brief interventions for alcohol use [1], has led to recommendations that SBIRT be a standard element of prenatal care [2]. However, studies comparing self-report of drug use to objective indicators show that underreporting is common [3-5], especially in settings where disclosure can have heightened negative consequences such as during pregnancy [4,6,7]. The disclosure of substance use during pregnancy can be both socially stigmatizing and increase the woman’s risk for potential legal consequences. Currently, 18 states view substance use during pregnancy as child abuse, and 15 states have laws stating that health care workers are mandated reporters for drug abuse during pregnancy. Laws such as these increase the social stigma and the internal shame and guilt women may feel. This in turn limits the proportion of women who are willing to disclose substance use to their providers, suppressing disclosure and impacting the health of them and their unborn child [8-10]. This underreporting is a substantial obstacle to proactive screening efforts that seek to identify at-risk pregnant and postpartum women, the majority of whom do not seek treatment for substance use [11].

Additionally, the implementation of SBIRT approaches has been challenging. First, there are considerable time, financial, and logistic obstacles to integrating screening and brief intervention programs into ongoing medical practice [12,13]. For example, one estimate suggests that conducting all recommended prevention-related activities would take a primary care physician 4.4 hours per working day [14]. This issue is exacerbated by the fact that such services are only recently and not consistently being reimbursed by third-party payers. Second, many medical professionals express discomfort with the screening and intervention process and report doubts about its effectiveness—even when voluntarily participating in a formal demonstration program [13]. This discomfort and skepticism may in part explain findings of very low levels of physician adherence to recommended brief intervention guidelines, even after training [12,14,15]. Training in brief approaches such as motivational interviewing is expensive, time-consuming, and may have modest or transient effects [16]. Technology provides an exciting option. It can be implemented consistently across patients, with minimal staff involvement, and conducted during natural waiting periods, integrating easily within the workflow of the clinic [17-19]. It has also been shown to improve disclosure of substance use in anonymous studies [20].

However, studies involving technology in health care settings often struggle with recruitment, particularly given time constraints on the part of clinic staff who must provide an initial introduction to the study. Typically, clinical trials are addressed via multisite trials using face-to-face recruitment. Despite being a time-tested gold standard, several limitations to this approach exist. First, the combination of a low base rate of substance use during pregnancy with high levels of underreporting makes recruitment lengthy and challenging even across multiple sites. Second, even multisite trials are only able to measure a limited range of participant characteristics specific to only a few geographic locations. Third, well-funded and tightly controlled trials often use methods (eg, a research assistant [RA] or study nurse) that do not readily translate to how the program could be implemented without research funding. Fourth, multisite research can also quickly become impractical if staffing each clinic with a full-time RA is required. There is increasing recognition of the need for highly pragmatic trials that take translational and implementation issues into account [21]. Research is therefore also needed on pragmatic methods for facilitating recruitment in these settings. The provision of flyers describing the study and allowing enrollment on the web is a possible solution, but relative recruitment rate for this approach, as compared to traditional approaches, is not known and is partly dependent on rates of technology ownership.

This study analyzes data exploring how to best leverage technology to identify risk during pregnancy, particularly whether different approaches in recruitment can increase disclosure. The study had 3 goals. The first goal was to obtain current substance use risk levels of women attending their prenatal care intake at a large Midwestern hospital’s outpatient clinic. The second goal was to compare the disclosure of substance use risk under 2 different recruitment strategies, in person versus via flyers, and determine recruitment rates for the 2 approaches. The last goal was to better understand the access and comfort of using smartphones and SMS text messaging for study participation. It was hypothesized that in-person recruitment would have a higher acceptance rate for study enrollment, but that participating in the study on their own device in the privacy of their home would increase disclosure.

## Methods

### Participants

Participants were 121 pregnant women attending a new pregnancy intake at an outpatient clinic that is part of a large health system in the Midwest. Eligibility criteria included being 18 years or older of age, understanding spoken English, and being pregnant with the intention to carry the pregnancy to term.

### Recruitment

Data collection began in September 2018 and concluded in May 2019. An RA was present at the clinic on 2 half days per week; during this time, willing participants were introduced to the RA by the nurse who was completing the intake with the patient. At all other times, clinic staff gave patients a flyer describing the study and provided a website (via QR code, along with a unique login ID) through which patients could enroll in and complete the study. The flyer was provided by intake nurses at the end of the appointment, and participants used their own device to complete the study.

### Procedures

Participants who enrolled in the study completed a series of screening questions regarding substance use before and during pregnancy, as well as questions related to demographics, general health, and technology access. Those recruited by the RA were given a tablet to complete the study at the clinic following their intake appointment. Those enrolling in the study via the flyer used their own device and completed screening at a time and place convenient for them. In addition, those who screened positive for any substance were offered within the app to participate in a subsequent extended assessment (duration of 10 to 20 minutes) with separate consent. The app would link participants to the next screening if they agreed to participate.

### Ethical Considerations

All procedures for the study were approved by both the university (#085518B3A) and health system (#12267) institutional review boards. The app used for data collection read aloud the consent form that explained to participants that the study has 2 parts. Electronic information sheets were used for the study. Participants agreed to participate in the study in the computerized questionnaire by clicking a box and then answering the questions. There were no physical copies of the consent form. Part I included content regarding broad health behaviors such as nutrition and sleep, as well as brief questions on smoking, alcohol, and marijuana use during the month before they became pregnant. For part II, patients who screened positive for smoking, alcohol, or marijuana use in the month before becoming pregnant were invited to complete a 15- to 20-minute survey asking additional questions about risk factors. This assessment included more sensitive information regarding substance use, traumatic experiences, partner violence, depression, and anxiety. Participants who completed part I received a US $10 Target gift card, and those who were eligible and completed part II received an additional US $20 Target gift card. No identifying information was collected until after participants completed the assessment items. Once participants completed the portions of the study they were eligible for, they were linked (within the survey) to a separate survey, where they entered their email or phone number to receive their gift cards and a copy of the consent form. The data participants gave in order to send the gift cards were kept in a separate password-protected spreadsheet from the rest of the data and were destroyed once the study was complete. Participants who completed the survey in the clinic received the consent form and gift card directly from the RA.

### Measures

All participants were asked to complete 47 items regarding alcohol, marijuana, and tobacco use before pregnancy and during the past month, as well as questions about pregnancy and general health. These items included, but are not limited to, the following:

1. The Tolerance, Annoyed, Cut Down, Eye-Opener (T-ACE) alcohol screen [22] is a 4-item alcohol risk screening questionnaire that asks about the amount of drinks to feel high, if people have annoyed you by criticizing your drinking, if you have ever thought you should cut down, or if you need to have a drink first thing in the morning. Scores of 2 or higher result in a positive screen.
2. The Wayne Indirect Drug Use Screener (WIDUS) [23] is a 6-item screening instrument that identifies risk for drug use in the perinatal period by asking about correlates of drug use without directly asking about use. Scores above 3 are considered positive. Examples of true or false questions include “most of my friends smoke cigarettes” and “I get mad easily and feel the need to blow off steam.”
3. The National Institute on Drug Abuse (NIDA) Quick Screen [24] consists of 4 questions asking respondents to indicate the frequency with which they had 4 or more drinks in a day, use of illegal drugs, use of prescription drugs for nonmedical reasons, or use of tobacco products in the past year. The alcohol and drug use items have been validated as single-item questionnaires [25,26]. These items were adapted to evaluate use in the past month rather than the past year and to include a separate item for cannabis use.
4. Participants were also given 4 technology questions regarding technology access and use (smartphone ownership, having an SMS text messaging plan, willingness to receive SMS text messages, and willingness to participate in research via a link sent to their phone).

### Statistical Analysis

Chi-square analyses compared differences between in-person– and flyer-based recruitment as well as differences in disclosure on the T-ACE, WIDUS, and each item of NIDA Quick Screen. Chi-square analyses used all available screening information from each participant. However, participants with missing items were dropped from that specific analysis. Two individuals had missing data for the NIDA Quick Screen binge drinking and tobacco questions. One person had missing data for the NIDA Quick Screen prescription drug and illegal drug use questions. There were no missing data for the T-ACE or WIDUS.

## Results

### Participant Characteristics

Study participants were primarily Black and African American (92/121, 76%) and had a mean age of 27.7 (SD 4.9) years (Table 1). Approximately half (66/121, 54.5%) of the participants had completed some education beyond high school.

**Table 1:** Participant race, ethnicity, and important demographic characteristics (N=121).

Characteristic		Values
Age (years), mean (SD)		27.7 (4.9)
**Race and ethnicity, n (%)**		
	Arabic	2 (1.7)
	Asian	4 (3.3)
	Black and African American	92 (76)
	Hispanic and Latino	5 (4.1)
	White	10 (8.3)
	Multiracial	4 (3.3)
	Chose not to answer	4 (3.3)
High school or General Educational Development test or higher, n (%)		66 (54.5)
Planned pregnancy, n (%)		45 (37.2)
First pregnancy, n (%)		34 (28.1)
Legally married, n (%)		31 (25.6)

### Risk Screen Positivity

Between-group differences in positivity rates were examined for 2 validated screening tools, the WIDUS and the T-ACE. A total of 20 (31%) out of 64 participants recruited through flyers screened positive for drug use risk on the WIDUS versus 9 (16%) out of 57 participants recruited at the clinic (*χ*^2^_1_=4.0; *P*=.05). Additionally, a total of 24 (38%) out of 64 women recruited through flyers screened positive for alcohol risk on the T-ACE versus 11 (19%) out of 57 participants recruited at the clinic (*χ*^2^_1_=4.9; *P=*.03; Table 2).

**Table 2:** Disclosure rates for substance risk indicators for in-person– and flyer-based recruitment methods.

Substance risk indicator	In person (n=57), n (%)	Flyer (n=64), n (%)
WIDUS^a^	9 (16)	20 (31)
T-ACE^b^	11 (19)	24 (38)
Past month alcohol binge	2 (4)	3 (5)^c^
Past month tobacco	8 (14)	11 (18)^c^
Past month opioid painkiller use	3 (5)	1 (2)^d^
Past month other drugs	14 (25)	9 (14)^d^

^a^WIDUS: Wayne Indirect Drug-Use Screener.

^b^T-ACE: Tolerance, Annoyed, Cut Down, Eye-Opener.

^c^n=62.

^d^n=63.

### Disclosure of Past Month Substance Use

**Figure 1:**
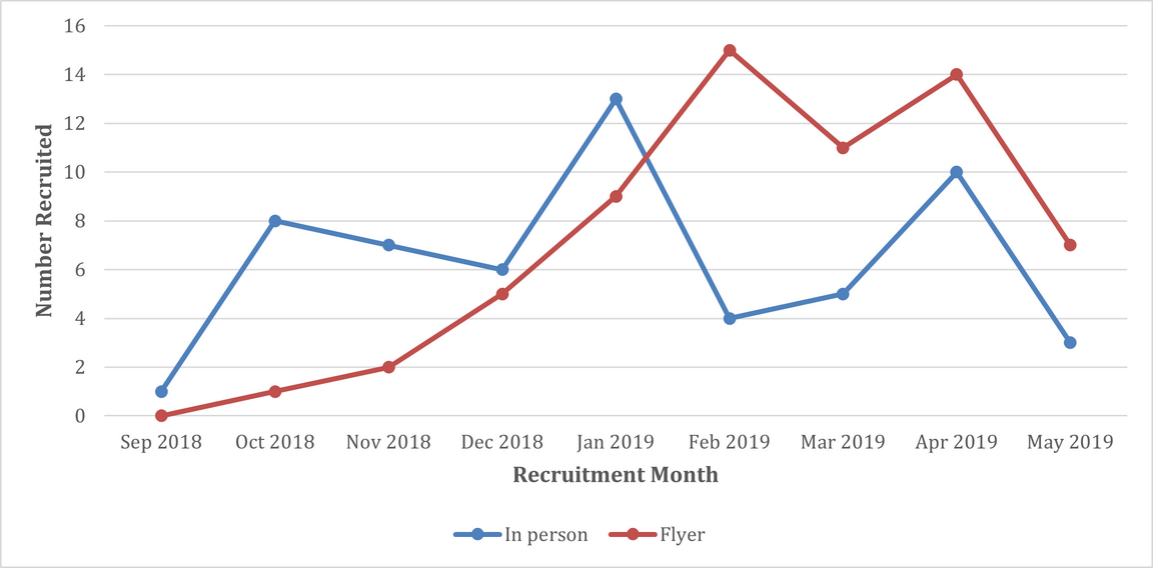
Recruitment rate comparison for the in-person and flyer clinic recruitment methods for patients attending prenatal care between September 2018 and May 2019.

Chi-square analyses compared the disclosure of substance use (on the NIDA Quick Screen) for participants recruited in person versus via the flyer. Questions included the frequency of binge drinking (4 or more drinks per day), tobacco use, prescription drugs for nonmedical purposes, and illegal drugs in the past month. Each NIDA Quick Screen item was treated as dichotomous reflecting either any or no reported use (Figure 1). There were no significant differences across groups in binge drinking (*χ*^2^_1_=0.1; *P*=.72), tobacco use (*χ*^2^_1_=0.3; *P*=.58), prescription drug use for nonmedical reasons (*χ*^2^_1_=2.0; *P*=.26), or illegal drugs (*χ*^2^_1_=2.0; *P*=.15).

### Recruitment Rates for Each Method of Recruitment

Of the 121 participants recruited overall, 57 were recruited directly by the RA, and 64 responded to the flyer. Nurses handed out 377 flyers resulting in 64 participants, representing 17% of those given the flyer. In contrast, of 109 patients completing a new pregnancy intake when the RA was in the clinic, 63 (57.8%) were introduced to the RA by the intake nurse, and 57 (91%) of the 63 agreed to participate and completed the screener (57/109, 52.3% of all available patients). Notably, flyer recruitment showed a stable increase over the course of the study (Table 2). This increase occurred following the introduction of a new approach, in which the RA and project coordinator began attending monthly staff meetings and updating nurses on the study progress, bringing in snacks, and building upon the relationships with the intake nurses in the clinic. This change in approach started in January, with an increase in nurse engagement and enthusiasm for the study happening in the next few months, resulting in an increase in flyer recruitment because many more were handed out. During the winter months of February and March, there were fewer intakes overall because of the weather. This decrease corresponds with an expected decrease in in-person recruitment during those months.

### Technology Accessibility and Willingness to Use for Participation

Of the 57 participants who were recruited in the clinic and used the tablet provided by the RA, 56 (98%) reported owning a smartphone, and 55 (96%) reported having an SMS text message plan on their phone. In total, 44 (77%) of these participants said that they would be willing to receive SMS text messages as part of a research study, and these participants also said that they would be willing to participate in additional surveys or programs if sent a link on their phone.

## Discussion

### Principal Findings

This study was set up to obtain substance use risk levels for women attending prenatal care at a large Midwestern hospital’s outpatient clinic, compare 2 different recruitment methods to examine which had higher recruitment rates and disclosure rates, and document participants’ access and comfort using smartphones and SMS text messaging for study participation. Recruitment via flyers distributed by health care staff was less efficient than when those same staff introduced patients to an on-site RA (57/109, 52.3% vs 64/377, 17% enrollment). However, participants in the flyer group were more likely to report substance use risk than those in the on-site RA group (20/64, 31% vs 9/57, 16% for the WIDUS and 24/64, 38% vs 11/57, 19% for the T-ACE). Most study participants owned a smartphone (56/57, 98%) and had an SMS text message package on their phone (55/57, 96%). Additionally, of the 121 participants, 94 (77.7%) were willing to receive SMS text messages or a link to further study participation on their devices.

Despite the lower overall enrollment compared to the on-site RA, the flyer approach requires less effort for medical staff and removes the need for a full-time RA at each study clinic. The flyer approach was also associated with greater disclosure on some measures of substance use. As is often the case, maintaining regular communication with clinic staff was particularly important in the flyer-based recruitment approach. These findings suggest that eligibility determination for substance use studies may be more successful and more representative (because of the wider possible reach with the same level of staffing) when using electronic screening with flyers rather than relying on full-time staff in the clinic. Flyer-driven recruitment appears to be a practical approach, given the high levels of access to technology among the pregnant urban participants and their willingness to use their personal devices for research. These latter findings are consistent with national survey data suggesting that smartphone ownership rates are high [27], and research suggesting that low-income patients are willing to use their own smartphone to participate in research [28].

### Limitations

The sample size, homogeneity of the sample, and preliminary nature of this research all contribute to clear limits in the generalizability of these findings. In addition, our sample size limited the ability to understand what variables may contribute to higher disclosure within the flyer recruitment group (ie, maternal age, parity, past substance use, or socioeconomic status).

### Conclusions

Using electronic methods for eligibility determination appears to facilitate disclosure and, thus, recruitment efficiency. Although flyer-based approaches are less efficient than in-person recruitment with an on-site RA, they may also facilitate disclosure and can allow cost-effective recruitment at multiple sites. Even low-income patients in perinatal settings are very likely to own a smartphone and be willing to use their own device to participate in research. This method can allow for larger study samples by decreasing the amount of money needed to support full-time RAs in each recruitment site. Instead, 1 RA could be used across multiple sites, which can free up funds for a larger number of site locations. This can allow for a wider variety of participants across the country and could be more translatable and easier to replicate or continue once funding ends.

## Abbreviations

NIDA: National Institute on Drug Abuse
RA: research assistant
SBIRT: screening, brief intervention, and referral for treatment
T-ACE: Tolerance, Annoyed, Cut Down, Eye-Opener
WIDUS: Wayne Indirect Drug Use Screener

## Data Availability

The data sets generated and analyzed during this study are available from the corresponding author on reasonable request.
